# Noncovalent Interaction of Tilmicosin with Bovine Serum Albumin

**DOI:** 10.3390/molecules23081915

**Published:** 2018-07-31

**Authors:** Beáta Lemli, Diána Derdák, Péter Laczay, Dorottya Kovács, Sándor Kunsági-Máté

**Affiliations:** 1 Department of Pharmaceutical Chemistry, Faculty of Pharmacy, University of Pécs, Rókus 2, H-7624 Pécs, Hungary; derdak.dia@gmail.com (D.D.); kunsagi-mate.sandor@gytk.pte.hu (S.K.-M.); 2 Department of General and Physical Chemistry, University of Pécs, Ifjúság 6, H-7624 Pécs, Hungary; kovacs7dorottya@gmail.com; 3 János Szentágothai Research Center, Ifjúság 20, H-7624 Pécs, Hungary; 4 Department of Food Hygiene, University of Veterinary Medicine, István 2, H-1078 Budapest, Hungary; Laczay.Peter@univet.hu

**Keywords:** veterinary drug, macrolide antibiotic, albumin-drug interaction, binding properties, thermal denaturation

## Abstract

Tilmicosin is a widely used antibiotic in veterinary applications. Its antimicrobial activity is ranged from Gram-positive and some Gram-negative bacteria towards activities against Mycoplasma and Chlamydia. Adsorption affinity of tilmicosin antibiotics towards bovine serum albumin was investigated by both spectroscopic (UV-vis, Photoluminescence) and calorimetric methods. The interaction was determined on the basis of quenching of albumin by tilmicosin. Results confirm noncovalent binding of tilmicosin on bovine serum albumin with 1:1 stoichiometry associated with pK = 4.5, highlighting possible removal of tilmicosin molecules from the albumin surface through exchange reactions by known competitor molecules. Calorimetric measurements have confirmed the weak interaction between tilmicosin and albumin and reflect enhanced denaturation of the albumin in the presence of tilmicosin antibiotic. This process is associated with the decreased activation energy of conformational transition of the albumin. It opens a new, very quick reaction pathway without any significant effect on the product by noncovalent binding the tilmicosin molecules to the protein molecules. Results highlight the medical importance of these investigations by considerable docking of the selected antibiotic molecules on serum albumins. Although the binding may cause toxic effects in living bodies, the strength of the binding is weak enough to find competitor molecules for effective removals from their surface.

## 1. Introduction

**Figure 1:**
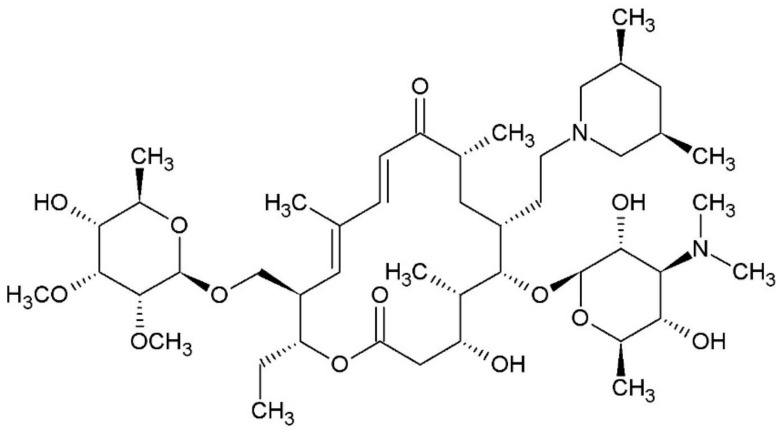
Chemical structure of tilmicosin.

Tilmicosin (Figure 1) is a broad-spectrum antibiotic for veterinary use. Effective applications are known against series of Gram-positive and Gram-negative bacteria and also antibacterial effects obtained for Mycoplasma and Chlamydia. Tilmicosin can be addressed as semi-synthetic macrolide extracted from the fermentation product of *Streptomyces fradiae*. It is recommended for treatment and prevention of pneumonia in cattle, sheep, ovine and swine and it is also approved for veterinary use in poultry. The residue of tilmicosin in the environment (e.g., animal food, milk and egg) is potentially noisome. Several methods are established to determine residues in both tissues and milk [1,2]. Furthermore, multiresidue methods for simultaneous analyzing of several different veterinary drugs including tilmicosin in meat, milk, egg and honey are also developed [3,4]. The binding properties of this veterinary drug to proteins highly affects its in vivo toxicity.

The most abundant serum proteins are the multifunctional serum albumins. These globular proteins are synthesized by mammals in their liver and they are highly similar in both sequence and structure [5]. Bovine serum albumin was extensively characterized for structural studies. It is a 66.4 kDa globular heart-shaped single-chain protein composed of 583 amino acid residues. It consists of three homologous domains (domains I–III), which are separated into nine loops (L1–L9) by 17 disulfide bridges [6]. The loops in each domain consist of a sequence of large-small-large loops that form a triplet. Each of the domains consists of two subdomains A and B. One of the fascinating properties of serum albumins is that they can form complex with several endogenous and exogenous molecules [7,8,9]. There are three major binding sites for these bioactive molecules on bovine (and human) serum albumin: Sudlow’s Site I and II on subdomain IIA and IIIA, respectively, and the recently described Hem binding site on subdomain IB [10,11]. It has great medical importance because albumin influences the tissue distribution and elimination of several compounds. Therefore, it is not surprising that albumins have been used for various biophysical, biochemical, and physicochemical studies as model proteins.

The binding of tilmicosin to bovine serum albumin was reported by electrochemical and UV-vis spectroscopic methods [12]. Due to several questions remained opened, in this study the interaction of tilmicosin (TIL) with bovine serum albumin (BSA) was investigated using optical spectroscopic (UV-vis, Photoluminescence) and Differential Scanning Calorimetric (DSC) methods.

## 2. Results and Discussion

### 2.1. Investigation of TIL-BSA Interaction by UV-Visible Absorption Spectroscopy

**Figure 2:**
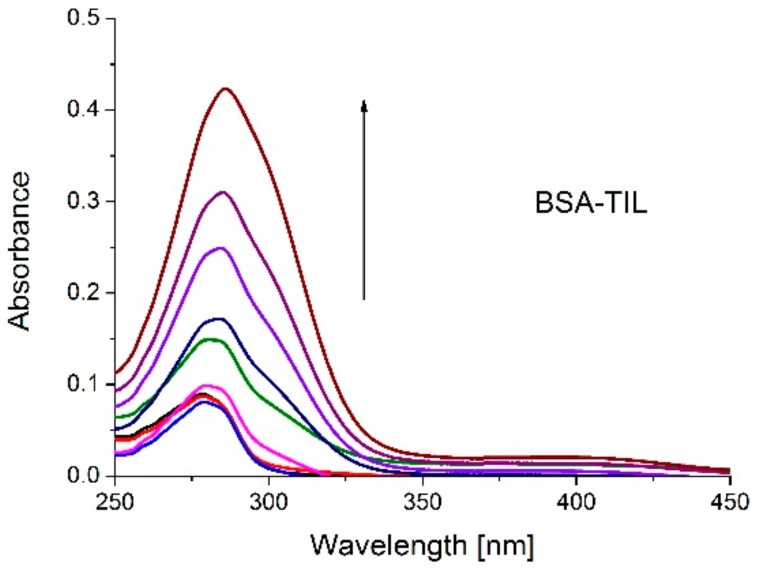
UV-vis absorption spectra of increasing TIL concentrations (0–15 μM) in the presence of 2 μM BSA.

UV-vis absorption measurement is a simple and easily accessible method to investigate the protein-drug interaction and their ground state complex formation. The UV-vis spectrum of BSA shows two absorption peaks: the strong peak at 210 and the weak peak at 280 nm are due to the absorption of the backbone and due to the aromatic amino acids (tryptophan, tyrosine and phenylalanine), respectively, while TIL has only one weak peak at 294 nm. To investigate the interaction of TIL with BSA, UV-vis absorption measurements were performed. Figure 2 shows the absorption spectra when increasing TIL concentrations (0–15 μM) were added to standard amount of BSA (2 μM). For further analysis of the changes in the microenvironment of the part of the protein, the measured data were corrected by subtracting the absorption spectra of TIL from TIL-BSA spectra. The aromatic amino acids of protein have prominent absorption bands in the region between 250 and 300 nm and this absorption peak of BSA centers at 279 nm. However, after addition of TIL the absorption intensity of the BSA increased, also the maximum peak position at 279 nm exhibited a bathochromic shift. These results verified weak molecular interaction between TIL and BSA. Furthermore, the red shift of the absorption spectrum indicates that the peptide strands extended a little, but the hydrophobicity increased [9], while the microenvironment around the aromatic amino acid residues changed during the complexation [13,14]. The interaction of the TIL induced a conformational change in BSA, which is primarily near to the aromatic region indicating hydrophobic interactions with aromatic amino acids of protein.

### 2.2. Investigation of TIL-BSA Interaction by Fluorescence Quenching Method

**Figure 3:**
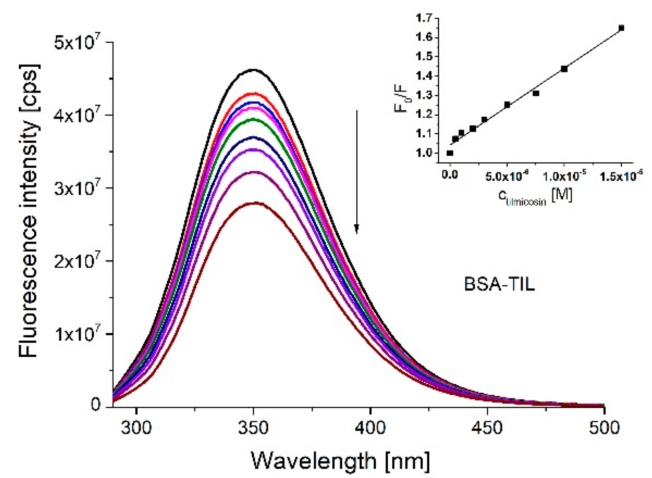
Fluorescence emission spectra of 2.0 μM BSA in the absence and presence of TIL with increasing concentrations (0.5, 1.0, 2.0, 3.0, 5.0, 7.5, 10.0 and 15.0 μM) in PBS (pH 7.4) buffer and the Stern-Volmer plot of the interaction (λ_exc_ = 280 nm, λ_em_ = 350 nm).

To confirm the interaction between TIL and BSA, fluorescence quenching studies were performed. To eliminate the inner filter effect, the fluorescence emission intensities were corrected using the absorbance data [15] and the corrected data were used for further calculations. The fluorescence emission spectra of BSA (2 μM) in the presence of increasing concentrations of TIL (0–15 μM) are shown in Figure 3. It can be clearly seen on the figure that the increased concentrations of TIL quenched the intrinsic fluorescence intensity of BSA. To verify the presence of static or dynamic quenching for the binding of TIL to BSA, the Stern-Volmer equation was applied:

(1) $$\frac{I_{0}}{I}=1+K_{q}\tau_{0}\left[Q\right]=1+K_{SV}\left[Q\right]$$

where *I_0_* and *I* are the fluorescence intensities of the BSA in the absence and presence of the quencher TIL, [*Q*] is the quencher concentration, *K_q_* is the quenching rate constant of the biomolecule, *τ_0_* is the average lifetime of the fluorescence and *K_SV_* is the Stern-Volmer quenching constant. The plot of the value *I_0_/I* versus the concentration of TIL shows linear correlation at the concentration range studied and indicating 1:1 stoichiometry of the complex formation (inset on Figure 3), and gave 3.14 × 10^4^ M^−1^ as *K_SV_* value. The average lifetime of the fluorophore is ~10^−8^ s [15] and the *K_q_* obtained from the Stern-Volmer equation is 3.14 × 10^12^ dm^−3^/mol·s. The maximum scattering collisional quenching constant is of various quencher ~2 × 10^10^ dm^−3^/mol·s for dynamic quenching [15]. Therefore, *K_q_* value supports a static quenching mechanism of the nonfluorescence complex formation between TIL and BSA.

### 2.3. Evaluation of the Association Constant Related to the Binding Process

The stepwise and overall binding (association) constants were calculated as described in details in our previous works [16,17]. Accordingly, the fluorescence emission data and UV-vis absorption data obtained during the experiments performed were evaluated by the Hyperquad2006 program (Ver. 3.1.60 Protonic Software, Leeds, England) package. In details, the stability constants associated with the complex formation of BSA with TIL were calculated by the Hyperquad2006 program package [18]. This code uses the following equations during calculation of the complex stabilities:

(2) $$pBSA+qTIL↔BSA_{p}TIL_{q}$$

(3) $$\beta_{pq}=\frac{\left[BSA_{p}TIL_{q}\right]}{{\left[BSA\right]}^{p}{\left[TIL\right]}^{q}}$$

where *p* and *q* reflect the stoichiometry associated with the possible equilibria in the solution. Then, all equilibrium constants are defined as overall association constants:

(4) $$BSA+TIL↔BSA\;TIL\;\beta_{1}=\frac{\left[BSA\;TIL\right]}{\left[BSA\right]\left[TIL\right]}$$

(5) $$BSA+2TIL↔BSA\;TIL_{2}\;\beta_{2}=\frac{\left[BSA\;TIL_{2}\right]}{\left[BSA\right]{\left[TIL\right]}^{2}}$$

(6) $$BSA+qTIL↔BSA\;TIL_{q}\;\beta_{q}=\frac{\left[BSA\;TIL_{q}\right]}{\left[BSA\right]{\left[TIL\right]}^{q}}$$

The relationship between the overall association constants *β_i_* and the stepwise association constants *K_i_* is defined as follows:

(7) $$\beta_{1}=K_{1};\;\beta_{2}=K_{1}\times K_{2};\;\beta_{q}=K_{1}\times K_{2}\ldots \times K_{q}$$

During the data evaluation, the stoichiometry associated with the minimum standard deviation between the measured and calculated spectral data was accepted as complex stoichiometry. Then, the stability constants were calculated by the model defined on this way.

Assuming 1:1 stoichiometry, the calculated association constant is 3.62 × 10^4^ M^−1^ and 2.80 × 10^4^ M^−1^ determined by Photoluminescence and Photometry measurements, respectively. These values are comparable, indicating similar stabilities and suggesting that the binding of TIL to BSA is stable and noncovalent. The calculated binding constant of TIL-BSA complex is consistent with the previously reported data of Bicer and Özdemir [12]. Moreover, strengths of TIL-BSA complexes are comparable with fluoroquinolones—BSA complexes [19]. According to the determinate data, it is assumable that the binding of TIL with BSA are probably biologically relevant interactions.

The concentration of serum albumin in blood is very high (40–50 g/dm^3^) as compared to the plasma concentration of the drugs used for therapeutic purposes; therefore, low drug: protein ratios are applied in the physiological system. For this reason, the bounded fraction of the added drug has been calculated from the association constant. Assuming a 1:1 stoichiometry, the binding reaction can be described as in Equation (4). In this special case, the concentration of the complex formed can be written as the function of the initial concentrations of the species interacted ([*BSA*]_0_ and [*TIL*]_0_):

(8) $$\left[TIL:BSA\right]=\frac{1}{2}\left\{\left({\left[BSA\right]}_{0}+{\left[TIL\right]}_{0}+\frac{1}{K_{1}}\right)\pm \sqrt{{\left({\left[BSA\right]}_{0}+{\left[TIL\right]}_{0}+\frac{1}{K_{1}}\right)}^{2}-4{\left[BSA\right]}_{0}{\left[TIL\right]}_{0}}\right\}$$

The concentration of drug bound to the protein [*TIL*]_b_ is equal with the [*TIL:BSA*] complex concentration. In this way, the association constant data was used to calculate the percentage of antibiotics bound. Considering 10 mg/kg body weight dosing of the antibiotics, the TIL:BSA ratio varies within range 1:3.7 … 1:6.0 and our results estimate that 54–67% of TIL bounded to BSA under the applied physiological conditions. It means that only a low fraction of added TIL is transported by BSA and a large amount of added drug remained and may not be available for pharmacological influence.

### 2.4. Thermodynamic Parameters

With the aim to investigate the effect of the temperature on TIL-BSA interaction, association constants were determined between 298.2 and 313.2 K. Table 1. summarizes the binding constant of TIL-BSA evaluated at different temperatures. These data show that in accordance with the static quenching process of the TIL-BSA complex formation, the higher temperatures promote the dissociation of the complexes. Furthermore, the temperature-dependence of the association constants offers possibility to determine the thermodynamic parameters of formation of the TIL-BSA complexes using the van’t Hoff equation:

(9) $$\ln K=-\frac{\Delta G}{RT}=-\frac{\Delta H}{RT}+\frac{\Delta S}{R}$$

In Equation (9), the Δ*H* and Δ*S* stand for the enthalpy and entropy changes of the complex formation, while Δ*G* is the Gibbs free energy change. *R* stands for the gas constant, while *T* is the temperature in Kelvin. The Δ*G* value calculated for 298.16 K was found to be −25.11 kJ/mol. This negative value highlights spontaneous character of the binding process between TIL and BSA at room temperature. Furthermore, this value reflects typical noncovalent interactions. Δ*H* and Δ*S* values of the TIL-BSA complex formations were −11.06 kJ/mol and 47.1 J/K·mol, respectively.

The nature of the binding forces were analyzed by Ross and Subramanian [20]. To do that, van der Waals interactions, hydrophobic forces, multiple hydrogen bonds or electrostatic interactions were considered. Generally, when van der Waals forces and hydrogen bond formation are the dominant roles during the interactions between albumins and other bioactive molecules, then both enthalpy and entropy changes have negative values. Considering the participation of the solvent molecules in the association processes, the positive entropy value reflects less-ordered structure of water molecules after formation of complexes. Also, according to the work of Ross and Subramanian, the electrostatic interaction usually signed by negative enthalpy change in combination with the positive entropy change. In our present case, the interaction seems to be an enthalpy-driven process, where the decomposition of (part of) the solvation shells and electrostatic forces may play a major role during the TIL-BSA complex formation.

### 2.5. Interaction of TIL with Porcine, Sheep and Goat Serum Albumins

Only human serum albumin and bovine serum albumin were extensively characterized for structural studies. However, the interaction of bioactive molecules with mammalian serum albumins has high biological importance in veterinary medicine, and in some cases the complex stabilities of albumin-ligand complexes showed marked species differences [21]. For this reason, the interaction of TIL was investigated with porcine (PSA), sheep (SSA) and goat (GSA) serum albumins as well. Similarly, to the TIL-BSA complexes, to quantify the stabilities of TIL-PSA, TIL-SSA and TIL-GSA complexes, fluorescence quenching studies were performed, and the association constants were evaluated by the Hyperquad2006 program package as described above. Although the association constants of the four tested albumins with TIL show some changes (Table 2), e.g., TIL-SSA complex has somewhat lower stability, the observed data support that the binding affinity of TIL toward the analyzed four different serum albumin species are almost the same.

### 2.6. Thermal Denaturation of BSA in the Presence of TIL

During DSC measurements, the change in the molar specific heat capacity (Δc_p_) of the system was measured as a function of the temperature. The resulted curve describes the temperature dependence of Δc_p_ having a maximum at a characteristic temperature (*T_m_*), which is related to the change in thermodynamic state (transformed and initial conformation) of the protein. The calorimetric enthalpy of the process can be calculated from the area under the curve, while baseline correction eliminates the different temperature dependence of the heat capacity of the products and reactants. The microenvironment affects the structure of the proteins, so their denaturation processes are greatly influenced by the reaction conditions used, which are also expressed in DSC thermograms. Therefore, the evaluated thermodynamic parameters and the characteristic temperatures depend on the concentration of the protein [22,23], the applied scanning rate [24], the solvent, the ionic strength [25], the pH [26], the fatty acid content [22], the presence of globulins [27], etc.

**Figure 4:**
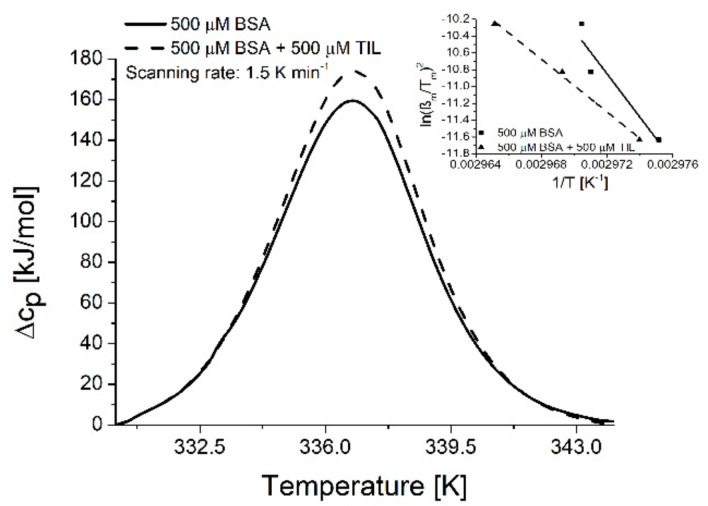
Representative DSC curves of BSA in the absence and presence of TIL and the Kissinger’s plots of the kinetic parameters.

Figure 4 reports the DSC curves of the investigated BSA in the absence and presence of TIL. A broad endotherm peak was obtained with a positive specific heat capacity change that can be assigned to the denaturing processes of the samples. During the endotherm process, the positive calorimetric enthalpy change means that more bonds of the reactants are dissociated than are formed in the products. This is not surprising because the conformation of native serum albumin is stabilized by several weak forces (hydrogen bond, van der Waals interaction, disulfide bridge). During the denaturation, these interactions are breaking down, and the unfolded protein cannot form similar bonds. The very high entropy change highlights that native structure of the BSA (folded state) is more ordered than its unfolded state. The scan rate-dependences of the enthalpy change, the entropy change and the transition temperature of BSA and TIL-BSA are reported in Table 3. Data shows that the presence of TIL did not affect significantly the enthalpy and entropy changes related to the thermal denaturation process of BSA. Thus, in the presence of TIL, the changes of Δ*H* and Δ*S* values suggest that the antibiotic drug does not have any stabilization or destabilization effect on the native structure of serum albumin. Nonetheless, the presence of TIL does not change significantly the *T_m_* value of the BSA, while the characteristic temperature of the transition depends on the applied scanning rate. Furthermore, to investigate the calorimetric reversibility of the thermally induced conformational change after a cooling to room temperature, the samples were heated again. Regardless of the presence of TIL, the conformational changes of the proteins could not be reproduced in the re-heated samples, thus the studied processes are irreversible. The reason is probably the aggregation of unfolded protein at higher temperatures.

Contrary to the difference-UV analysis, the discrimination of the unfolding and the aggregation phases of the denaturation process could not be obtained by differential scanning calorimetry analysis [28]. Accordingly, DSC measurements followed the Arrhenius equation and this property enables evaluation of the measurements using the Kissinger method. Therefore, the dependence of the thermal transition on the scan rate was determined and samples were scanned with the rate 1.0 K/min, 1.5 K/min, and 2.0 K/min. The activation energies were calculated by the Kissinger method [29],

(10) $$ln\left(\frac{\beta_{m}}{T_{m}^{2}}\right)=ln\frac{AR}{E_{a}}-\frac{E_{a}}{RT_{m}}$$

where *β_m_* is the scanning rate, *T_m_* is the transition temperature, *A* is the Arrhenius pre-exponential factor, *R* is the gas constant and *E_a_* is the activation energy. Figure 4 inset shows the Kissinger’s plots of the samples containing 500 μM BSA and samples containing 500 μM BSA + 500 μM TIL and Table 3 summarizes the activation energies related to the thermal denaturation of BSA in the absence and presence of TIL. As data demonstrate, the activation energy decreases in the presence of TIL, indicating that the presence of TIL promotes the thermal denaturation of BSA. The kinetic properties of the thermal denaturation process of BSA are significantly influenced by TIL. Hence, the increased entropy related to the interaction of antibiotic molecule with the serum albumin molecule observed from Photoluminescence measurements is not probably due only to decomposition of the solvation shells of the interacting molecules but is also due to the induced local change in the conformation of BSA. It is presumable to assume that this local conformation change of the albumin pre-organizes the molecule towards unfolding and opens a new reaction channel with lower activation energy. This can explain the more rapid unfolding of BSA in the presence of TIL.

## 3. Materials and Methods

Bovine serum albumin (BSA), sheep serum albumin (SSA), goat serum albumin (GSA) and porcine serum albumin (PSA) were purchased from Sigma-Aldrich (Budapest, Hungary). Tilmicosin (TIL) was obtained from Lavet Pharmaceuticals Ltd. (Budapest, Hungary). All the other analytical grade chemicals were purchased from VWR International Ltd. (Debrecen, Hungary). Phosphate-buffered saline (PBS) contained NaCl (137 mmol/L), KCl (2.7 mmol/L), NaH_2_PO_4_ (8 mmol/L), K_2_HPO_4_ (1.5 mmol/L) was prepared in ultrapure water (pH 7.4).

The UV-vis spectra were recorded by Specord Plus 210 spectrophotometer (Analytik Jena, Jena, Germany) equipped with a temperature controller (±0.1 K). For data collection, photon counting method with 0.1 s integration time was used and 2 nm bandwidths set and a quartz cuvette with 1.0 cm thickness were used. The measurements were carried out at 298.2 K.

Highly sensitive Fluorolog τ3 spectrofluorimeter (Jobin-Yvon/SPEX, Longjumeau, France) was used to investigate the fluorescence spectra of the different solutions. For data collection, photon counting method with 0.1 s integration time was used. Excitation and emission bandwidths were set to 4 nm. A 10 mm thickness of the fluorescent probes with right-angle detection was applied. The measurements were carried out at 298.2 K and the temperature-dependent measurements were also performed at different temperatures: 298.2 K, 301.2 K, 304.2 K, 307.2 K, 310.2 K and 313.2 K.

Differential scanning calorimetric (DSC) measurements were performed with a highly sensitive nano-II-DSC 6100 (Setaram, Caluire-et-Cuire, France) instrument. The calorimeter was configured with a platinum capillary cell (volume = 0.299 mL). Samples were pressurized to (3 ± 0.02) × 10^5^ Pa during all scans. Exploiting the reversible character of the binding reaction, cyclic scans were carried out within 293.15–353.15 K temperature range with the scanning rate of 2.0 K/min, 1.5 K/min, and 1.0 K/min forward and backward. Excess heat capacity, enthalpy change, entropy change, and transition temperature were calculated by subtraction of the baseline using the software of the calorimeter (CpCalc/SETARAM, 2003).

All of the data presented in the manuscript are the mean of 3 to 7 independent measurements with the related standard deviation.

## 4. Conclusions

Affinity of tilmicosin antibiotics towards bovine serum albumin was investigated by spectroscopic (UV-vis, Photoluminescence) and calorimetric methods. The interaction was determined on the basis of fluorescence quenching of bovine serum albumin by tilmicosin. Results confirm noncovalent binding of tilmicosin to bovine serum albumin with 1:1 stoichiometry. The binding constants observed in phosphate buffer (pK = 4.5) are in good correlation with the data obtained earlier in Britton-Robinson buffer solutions using electrochemical methods [12]. Furthermore, the determined stability constant is in the range of values of the fluoroquinolone antibiotic drugs with veterinary importance reported in the literature [9,19]. These results highlight possible removal of tilmicosin molecules from the bovine serum albumin surface through exchange reactions by several known competitor molecules. Calorimetric measurements have confirmed the weak interaction between tilmicosin and bovine serum albumin and reflect enhanced denaturation of the bovine serum albumin in the presence of tilmicosin antibiotic. This process is associated with the decreased activation energy of conformational transition of the albumin. The considerable binding of the tilmicosin molecules on serum albumins highlights the significant role of the albumins in the transport of this drug during medical applications. Although the binding may cause toxic effects in living bodies, the strength of the binding is weak enough to find competitor molecules for effective removals from the albumins’ surface.

## Figures and Tables

**Table 1:** Binding constants of TIL-BSA complexes at different temperatures.

	298.2 K	301.2 K	304.2 K	307.2 K	310.2 K	313.2 K
**logK** (±SD)	4.56 ± 0.01	4.53 ± 0.03	4.51 ± 0.02	4.50 ± 0.01	4.48 ± 0.01	4.46 ± 0.02

**Table 2:** Binding constants of TIL-serum albumin complexes.

	BSA	PSA	SSA	GSA
**logK** (±SD)	4.56 ± 0.01	4.52 ± 0.02	4.36 ± 0.05	4.53 ± 0.03

**Table 3:** Scan rate-dependence of transition temperature and the related enthalpy change, entropy change and the activation energies associated with the unfolding of BSA in the absence and in the presence of TIL.

500 μM BSA	Scanning Rate (K/min)		
	1.0	1.5	2.0
∆*H* (kJ/mol)	807 ± 43	747 ± 10	798 ± 27
∆*S* (J/Kmol)	2402 ± 128	2245 ± 28	2371 ± 80
*T_m_* (K)	336.1 ± 0.1	336.6 ± 0.2	336.7 ± 0.0
*E_a_* (kJ/mol)	2141		
**500 μM TIL + 500 μM BSA**	**Scanning Rate (K/min)**		
	1	1.5	2
∆*H* (kJ/mol)	693 ± 63	766 ± 28	791 ± 111
∆*S* (J/Kmol)	2050 ± 187	2259 ± 84	2343 ± 329
*T_m_* (K)	336.3 ± 0.2	336.8 ± 0.1	337.3 ± 0.2
*E_a_* (kJ/mol)	1304		
